# Chemical improvement of soluble rocks: Foundation of Mosul Dam as case study

**DOI:** 10.1038/s41598-024-64593-5

**Published:** 2024-06-19

**Authors:** Aram Aziz, Abbas Soroush, Seyed Mohammad Fattahi, Seyed Mohammad Reza Imam, Mehrdad Ghahremani

**Affiliations:** 1 Department of Civil and Environmental Engineering, Amirkabir University of Technology, Tehran, Iran; 2 Faculty of Engineering, Koya University, Erbil, KRI Iraq

**Keywords:** Mosul Dam foundation, Dissolution, Chemical improvement, Gypsum, Anhydrite, Civil engineering, Geology

## Abstract

The dissolution of soluble rocks (gypsum/anhydrite) beneath the Mosul Dam by water seepage has been observed upon the initial impoundment; consequently, several sinkholes have been manifested in the vicinity of the dam site. Traditional grouting has been envisaged as a potential remedy; however this measure has not eradicated the problem. The main purpose of this study is to overcome the solubility of the gypsum/anhydrite rocks using chemical grouts. Rock samples were acquired from the Fatha Formation outcrop and problematic layers of brecciated gypsum situated at varying depths beneath the Mosul Dam. Two commercially available liquid polymers, polyurethane (PU) and a mixture of acrylic and cement (ARC) were used to investigate their sealing performance in halting of the solubility of the rocks (gypsum/anhydrite). To simulate the dissolution phenomenon under the influence of artificial hydraulic pressure of the dam and the water flow in its abutments, two distinct laboratory models were devised. The outcomes from the experimental study on both untreated and treated samples revealed that the acrylic-cement composite (ARC) and polyurethane (PU) are influential polymers in halting the solubility of the gypsum rock samples under both factors of water pressure and high-velocity water flow.

## Introduction

Mosul Dam has been built on a problematic foundation, consisting mainly of highly jointed and karstified alternating beds of limestones, gypsum, and marls^1^. The dissolution of the soluble rocks (gypsum/anhydrite) by water seepage in the dam foundation has been observed upon the initial impoundment. As a result, several sinkholes in the vicinity of the dam site have been recorded^2,3^.

Similar to the Mosul Dam, several dams in different parts of the world have suffered from dissolution of the soluble rocks in their foundation or abutments. Kama Dam, in Russia^4^, Moses-Saunders Dam in Canada/USA^5^, La Loteta Reservoir in Spain^6^, Nurek Dam in Tajikistan^4^, and Huoshipo Reservoir in Chine^7^ are the most prominent instances.

The soluble rocks (Gypsum/Anhydrite) are generally monomineral rocks characterized by their soft and highly soluble minerals, low strength, highly deformable, and time dependent behaviors (creep, expansion, etc.)^8^. For many decades, the dissolution phenomenon of these types of rock has been a major theme in scientific inquiry. The initial inquiry into rock dissolution dates back to Nernst^9^, followed by Maslov and Naumenko^10^, Fabuss et al.^11^, Wigley^12^ Milanović et al.^13^ and Shearman and Mossop^14^

The kinetics of the dissolution of gypsum and anhydrite is shown by Eq. (1)^15^.

1 $$\frac{\text{dm}}{{\text{dt}}}\text{=KA(}{\text{C}}_{\text{s}}\text{-C}{)}^{\text{n}}$$

where, m is the mass of the dissolved CaSO_4_ at time t (s), *K* is the constant of dissolution rate, A is the area of sulfate rock subjected to the water flow, C_s_ is the rock solubility or saturated concentration (kg/m^3^), C is the concentration of dissolved rock at time t, and n is 1 for gypsum and 2 for anhydrite.

Equation 2 describes the concentration change rate of CaSO_4_ in water^16^.

2 $$\frac{\text{dc}}{{\text{dt}}}= \text{K} \frac{\text{A}}{\text{v`}}\text{(}{\text{C}}_{\text{s}}\text{-C)}$$

where, *v*` is the volume of the solution.

Upon integration of Eq. (2), Eq. (3) is derived, illustrating the relationship between the concentration and time.

3 $${\text{Ln}}\left(\frac{{\text{C}}{\text{s}}}{{\text{C}}{\text{s}}\text{-C}}\right)\text{=}\frac{\text{KA}}{\text{v`}}{\text{t}}$$

In laminar flow situation there is a direct correlation between K and V. As the flow transitions from laminar to turbulent, there is an exponential increase in the dissolution rate^15^.

Despite the vast impact of halting gypsum dissolution under hydraulic structures through chemical grouts, the scope of the research addressing this area remains surprisingly sparse. For example, Nikolaev ^17^ has investigated the impact of chemical solutions on gypsum stability. According to the results, the diluted oxaloaluminosilicate and sodium silicate liquids lead to significant reductions in discharge over time, sometimes completely stopping seepage in the experiments. Previous investigations have predominantly focused on the mechanical improvement of gypseous soils ^18–20^, leaving a conspicuous gap in the chemical grouting of soluble rock to halt their solubility. On the other hand, there is no published research that has previously applied the current study's materials in the treatment of soluble rocks. Considering this gap, the primary objective of this research is to evaluate the efficacy of two commercial liquid polymers, including polyurethane (PU) and acrylic-cement mixture (ARC) in controlling the dissolution of the soluble rock samples. For this purpose, several gypsum samples were collected from the Fatha Formation outcrop and problematic layers of brecciated gypsum (GB0, GB1, GB2, and GB3) at different depth of the Dam foundation. The selection of these gypsum rocks to treat stems from their resistance to cementitious grout and preventing the formation of a strong physical bond (adhesion) between the gypsum rock and the grout. This defect has been noted in the protective measures of the Mosul dam foundation^21^. The selection of these gypsum rocks is due to their resistance to cement grout and prevent the formation of a strong physical bond (adhesion) between them^21^. To simulate the dissolution phenomenon under the influence of artificial hydraulic pressure of the dam, the water flow in its abutments, two distinct devices, high velocity-based and pressure-based apparatuses, were utilized for conducting the dissolution tests to assess the impact of the used polymers on the rock samples solubility.

### Short background on chemical grouting

Chemical grouts were created in the early 1900s to reinforce and control water flow in geological units with small pores which are unsuitable to be grouted by regular cement grouts. Chemical grouting entered its contemporary phase in the early 1950s, propelled by advancements in polymer industries^22^. Polymers are generated by chemically reacting monomers which are small molecular compounds. They typically consist of multiple structural units joined together by covalent bonds^23^.

Grouts may be categorized as the traditional (i.e., cement, fly ash, lime, bitumen, etc.) and non-traditional (e.g., polyurethane, acrylate, sodium silicate)^24^. The main advantages of the polymer grouts are their high penetrability, low viscosity, controllability of their gelling time and high adhesivity. These characteristics render them to consider as viable alternatives to traditional cement^25,26^. In recent years, chemical grouting materials such as polyurethane and silica sol^27^, butyl acrylate and styrene^28^, sodium silicate^29^, and polyacrylamide^30^ have been utilized in soil remediation.

PU is the product of combination of polyol (-OH) and isocyanate (-NCO). Depending on its response to water, PU is classified into hydrophobic and hydrophilic. Hydrophobic absorb minimal additional water, while, hydrophilic grouts assimilate considerable amounts of water into their chemical structure^31^.

Several studies^32–37^ have investigated different aspects of PU such as, its durability, tensile properties, mechanical properties, and toxicity.

Acrylic polymers or thin film coatings represent another category of polymers. In recent years, the use of thin film coatings has jumped into the engineering field, particularly in soil and rock stabilization^38^, owing to their broad range of features like flexibility and excellent adhesion, mechanical strength, scratch resistance, and thermal stability^39,40^.

In their study, Chhun et al.^40^ have concluded that the acrylate-cement grout played a crucial role in enhancing the mechanical strength of silty sand. Kolay et al.^26^ have investigated the impact of liquid acrylic polymer on geotechnical properties of fine grained soils. They have noted that the utilized grout does not offer a significant impact on the Atterberg limits and unconfined compressive strength; however, it influences the CBR results.

## Mosul Dam, its foundation, and a brief history of sinkholes development

**Figure 1:**
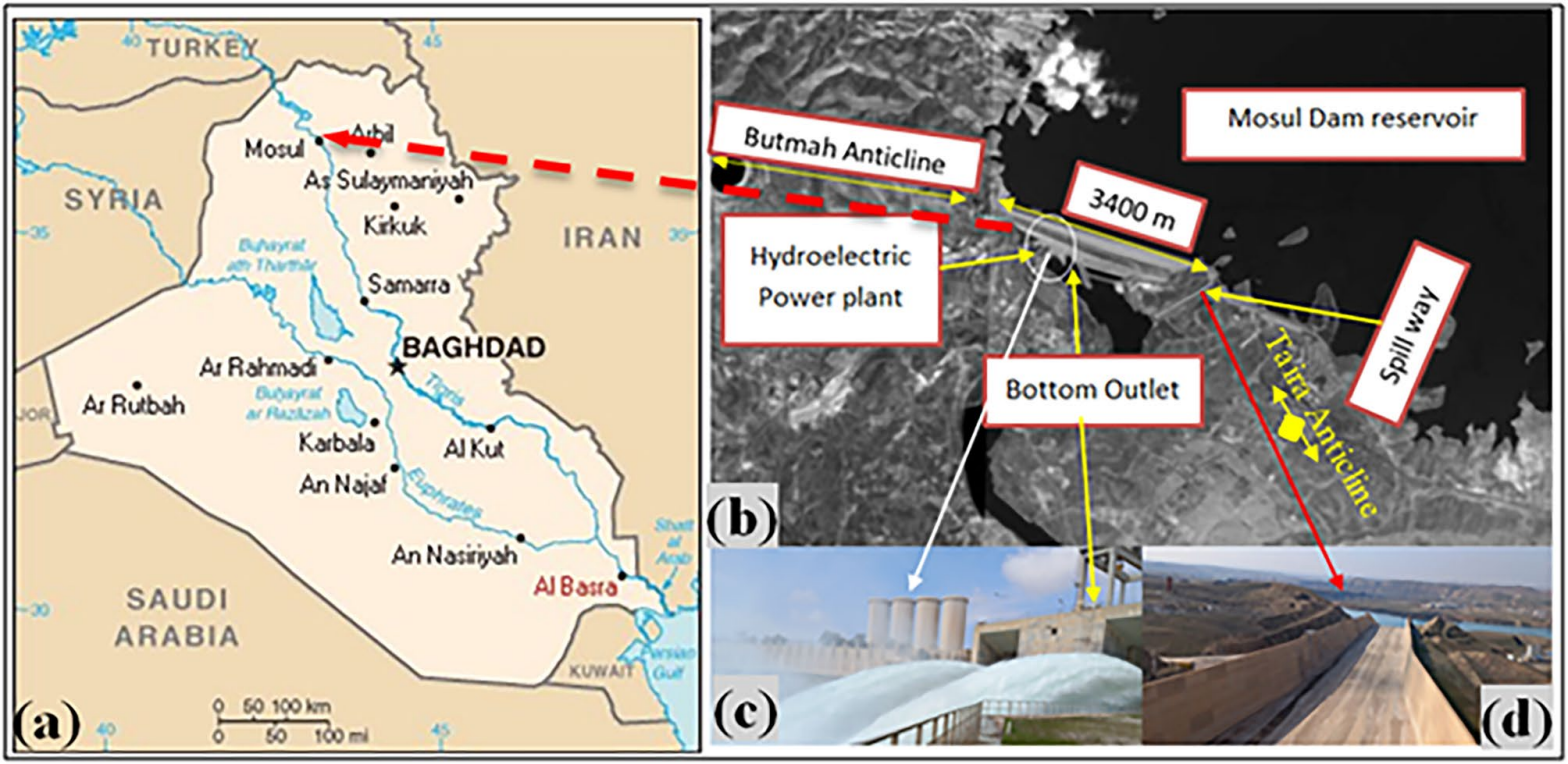
(**a**) Mosul Dam location https://www.worldometers.info/maps/iraq-map/ , (**b**) aerial view of the Mosul Dam (USACE) (**c**) hydroelectric and bottom outlet, (**d**) spillway.

**Figure 2:**
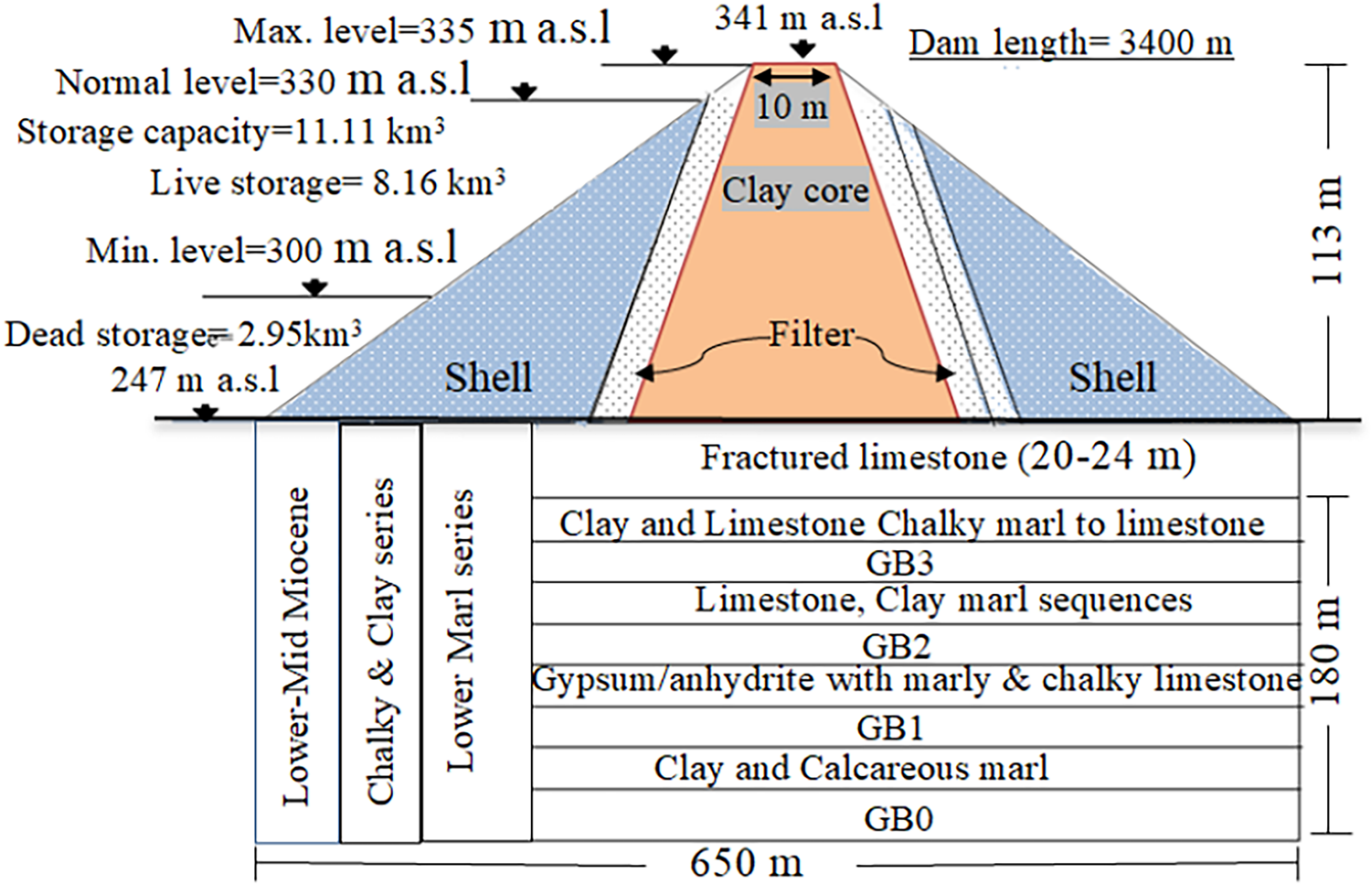
Schematic representation of the geometry of the Mosul Dam and the stratigraphic column of its foundation.

Mosul Dam is a multipurpose earth fill embankment located on the Tigris River in northwest of Mosul city in northern Iraq. Figure 1 shows the Mosul Dam location. This hydraulic structure with a storage capacity of 11.11 billion m^3^ started operating in 1986, and from then on, it struggled with seepage along with the dissolution of the gypsum beds in the dam foundation and its abutments^3,41^. From structural geology standpoint, this project is situated between two anticlines, Butmah west anticline on the right abutment and the Taira anticline on the left abutment^1,21^. Two prominent geological formations exist at the dam site and reservoir, including Fatha Formation (Middle Miocene) with lithology of marl, limestone, and gypsum sequences, and the Euphrates-Jeribe Formation (lower Miocene) comprising limestone, dolostone beds, and marl^1,3,42,43^. Figure 2 represents the schematic diagram of the dam cross section and its foundation.

Due to the presence of soluble rocks, karstification is the most significant geological phenomenon at the Mosul Dam site. Sinkholes which have developed in both the dam site and the reservoir area are the most prevalent feature of this phenomenon. The existence of brecciated gypsum (GB) at different depths of the dam foundation is another feature^44–46^.

In 1986, a number of sinkholes were recognized on the right bank, approximately 150 m away from the dam contact with the right abutment^41^. Between 1992 and 1998, four sinkholes in a linear arrangement parallel to the dam axis developed^1^. Also, in February 2002, a large sinkhole with 15 m depth and width appeared just 150 m downstream of the dam toe at the left bank^21^. These sinkholes prove continuous gypsum dissolution in the dam foundation and abutments.

## Testing materials and methods

### Gypsum rocks and sampling

**Figure 3:**
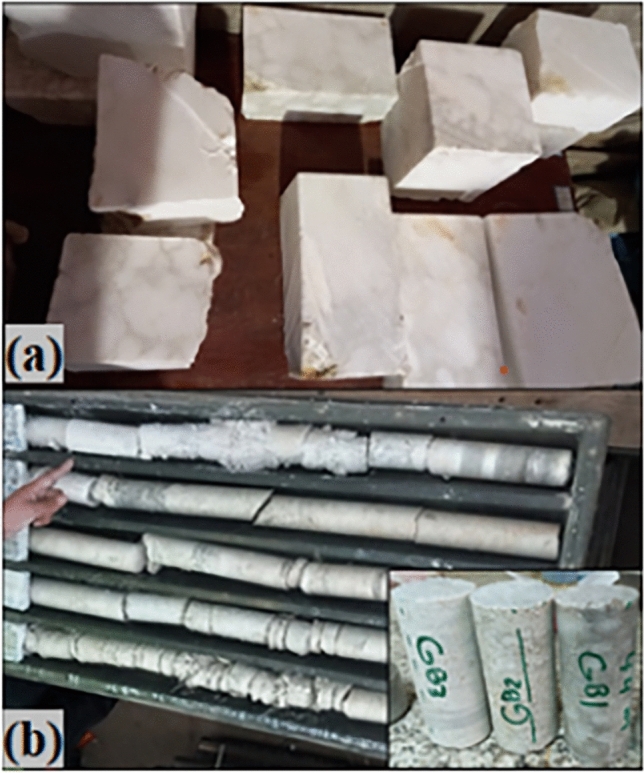
Gypsum rock samples, (**a**) blocks of surface rocks, (**b**) core samples taken from boreholes.

In this research, the sulfate rock samples were collected from two sources: the Fatha Formation outcrop or (surface samples-S) and boreholes within problematic layers of the Mosul Dam foundation (G). Surface samples were collected in large pieces and then cut into blocks (Length = 25 cm, Width = 15 cm, variable height of 4–6 cm) for dissolution test under high-velocity water flow, simulating surface dissolution in the dam abutments Fig. 3a. The core samples from boreholes Fig. 3b, labeled as G1 (from GB1, depth of 44m), G2 (from GB0, depth of 76 m), G3 (from GB3, depth of 94 m), and G4 (from GB2, depth of 130 m) were retrieved from the Mosul Dam Core Samples Conservation Warehouse. These core samples with 5 cm diameter were trimmed to achieve a length-to-diameter ratio of 2.3. They were then employed to quantify their solubility under pressurized water flow (400 kPa), simulating rock dissolution in the dam foundation.

The remaining fragments from each sample were reused for their chemical analysis. To identify the mineral composition of the rock samples, two methods were employed: microscopic analysis (thin sections) and X-ray diffraction (XRD).

The microscopic analysis revealed that the predominant components of both type rock samples were gypsum and anhydrite.

**Figure 4:**
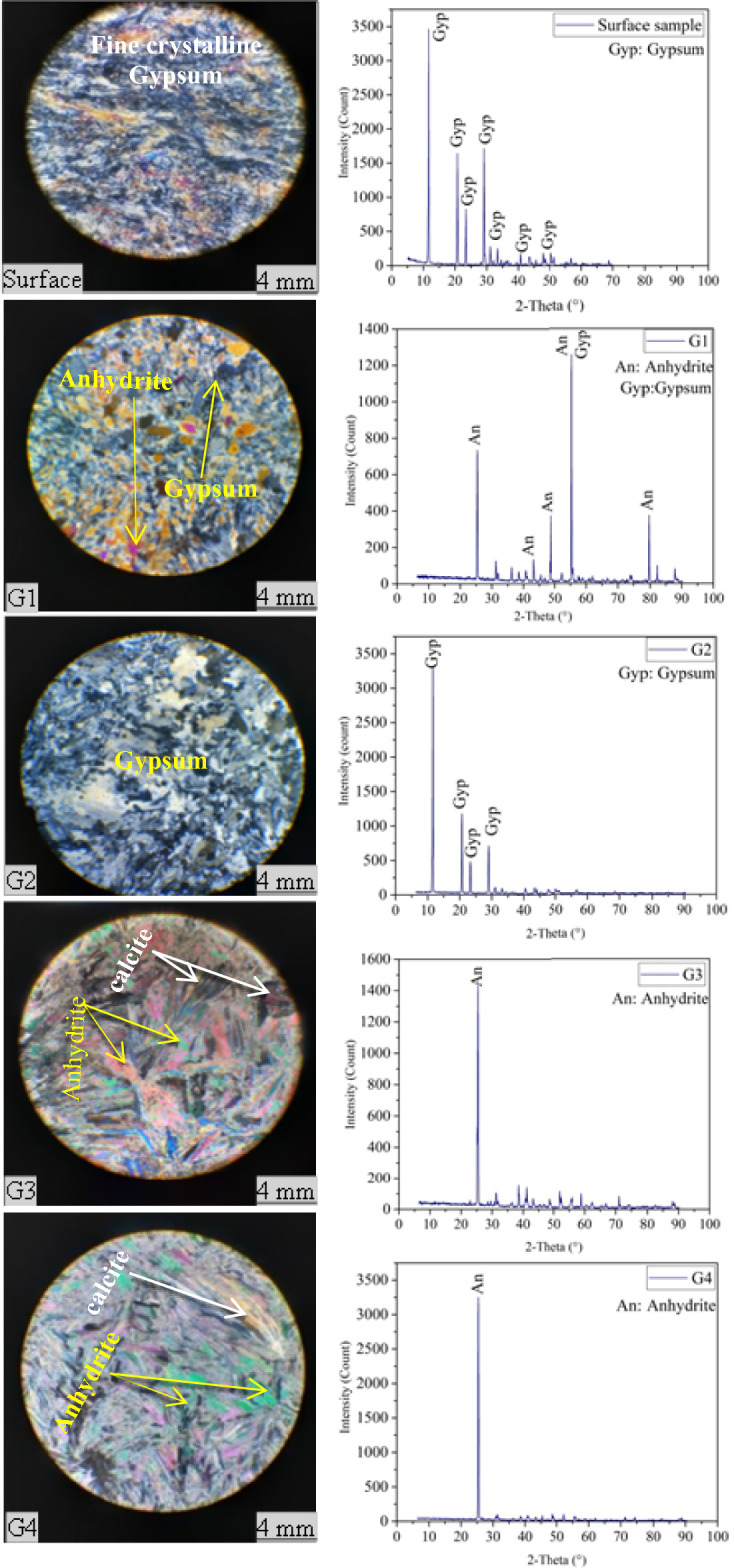
Microscopic images and corresponding XRD analysis of the surface and core samples.

On the other hand, X-ray diffraction (XRD) analysis revealed that gypsum with 2ϴ peak of 11.6, 20.65, 23.28 and 29.05 constitutes the primary component of the surface specimens and G2 Koukouzas and Vasilatos^47^ achieved a similar result. While G3 and G4 samples were recognized as anhydritewith 2ϴ peak of 25.45 and accessory minerals of calcite with 2ϴ peak of 40, 43 and 48. A similar result was achieved by Al-Jaroudi a^48^. The G1 sample exhibited a mixture of both gypsum and anhydrite with 2ϴ peak of 25.5 and 55.5, respectively. Figure 4 shows the microscopic images and XRD patterns of the samples.

### Acrylic-cement grout (ARC)

The emulsion liquid acrylic polymer utilized in this study was mixed with type II Portland cement and aluminum sulfate accelerator (1.2 Molarity) in different proportions of (Acrylic: Cement: Al_2_SO_4_ by weight = 5:0.5:0.5, 5:0.75:0.5, 5:1:0.5 and 5:1.5:0.5). The cement contents of the grout were varied, while the polymer weight and the aluminum sulfate proportions were kept constant. Choosing a 0.5 ratio of aluminium sulfate (1.2 M) was based on the consideration that more than that ratio would have lowered the initial fluidity of the grout to a great extent. The physical and chemical specifications of the liquid polymer are presented in Table 1.

**Table 1:** Physical and chemical properties of acrylic and PU.

Property	Acrylic	Seal Boss1510	Accelerator
Appearance	Milky	Amber	Clear
Density, g/cm^3^	0.99	1.12	0.93
Viscosity, cps	3	160–250	20
Solubility in water	Dilutable	Not	Not

### Polyurethane (PU)

In this study, the hydrophobic foam (Seal Boss 1510) based on diphenylmethane diisocyanate MDI polyurethane was utilized. Its gelling time was adjusted by incorporating a 15 × accelerator. The physical and chemical specifications of the PU are given in Table 1.

The reaction of the foam varies based on the accelerator quantity. The higher accelerator percentages lead to increased reactivity, shorter hardening time, more expansion, and lower density, this was confirmed by^49^.

To achieve the polyurethane foam with different densities and gelling times, the range of accelerator quantities were varied with 5 percent interval from 5 to 25%.

### Sample treatment

**Figure 5:**
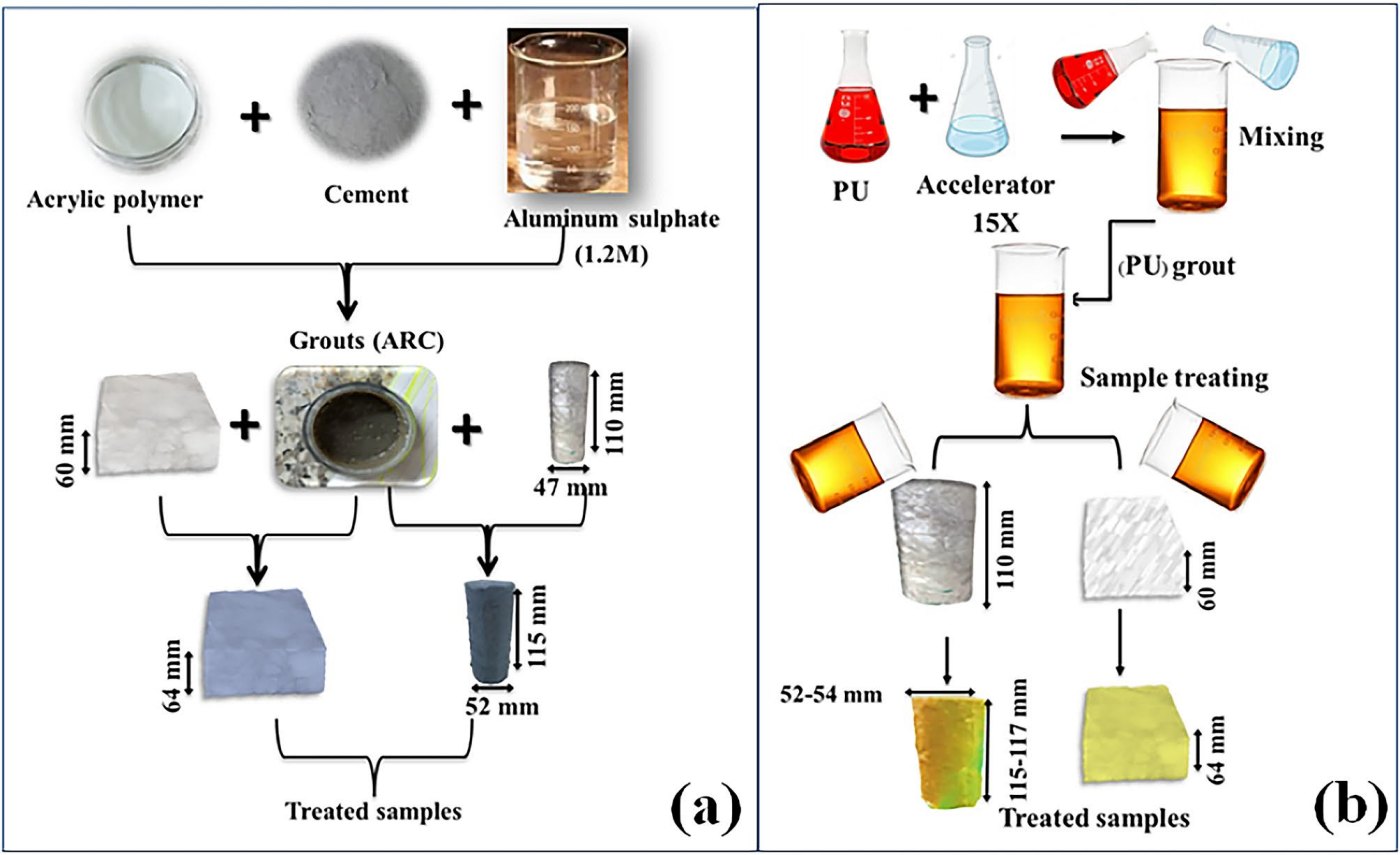
Schematic illustration of the sample treatment procedure, (**a**) ARC and (**b**) PU.

The treatment procedure of the samples with ARC is represented in the Fig. 5a. In the treatment of the surface samples, all surfaces of the sample were coated with ARC. At first, all surfaces of the sample except one surface were coated (painted) by the ARC grout. This created a uniform layer with 2 mm on the coated surfaces. After setting this layer, the last surface of the sample was coated with 2 mm thick layer of ARC. Finally, the coated sample was left at laboratory temperature (25 ± 2 °C) for 24 h and then subjected to a dissolution test at 0.3 m/sec water flow velocity.

For the treatment of the core samples with ARC, initially, the surfaces of the sample along its height as well as one of its ends were coated with a uniform layer of the ARC. This caused an approximate 4 mm increase in the sample diameter and 2 mm in its height once the ARC had solidified (a caliper was used to measure the diameter of the treated sample). After drying this part, the other end of the core sample was coated with 2 mm thick layer of ARC; thus, the sample's height increased by 4 mm. After setting of the last part, the treated core sample was left for 24 h in the room temperature. Finally, the solubility of the ARC treated core samples were investigated under constant pressure of 400 kPa.

The total number of ARC treated samples was eight samples. Four ARC treated surface samples (S) were labeled as (S-ARC 5:0.5, S-ARC 5:0.75, S-ARC 5:1 and S-ARC 5:1.5), and four ARC treated core samples (G2) were labeled as (G2-ARC 5:0.5, G2-ARC 5:0.75, G2-ARC 5:1 and G2-ARC 5:1.5).

Concerning the treatment of the samples with PU Fig. 5b, the same approach used for ARC treated samples was used to treat the samples with PU. However, due to the variation of PU expansiveness with the change in accelerator amount, it was slightly challenging to treat the samples with PU. Therefore, the PU thickness on the surface of the treated samples for different tests with different accelerator ratio was not uniform. PU with a greater accelerator ratio is more expansive and lower density. The total number of PU treated samples for the surface (S) and core samples (G) were 10. Five samples of each type were treated with different accelerator ratio (S-5% acc to S-25% acc) and (G2-5% acc to G2-25% acc).

It is worth mentioning that the S- treated samples were tested under high-velocity water flow and the G2-treated samples were tested under a pressure of 400 kPa, and all experiments were performed at a temperature of 25 ± 2 °C.

### Dissolution test using velocity-base apparatus

**Figure 6:**
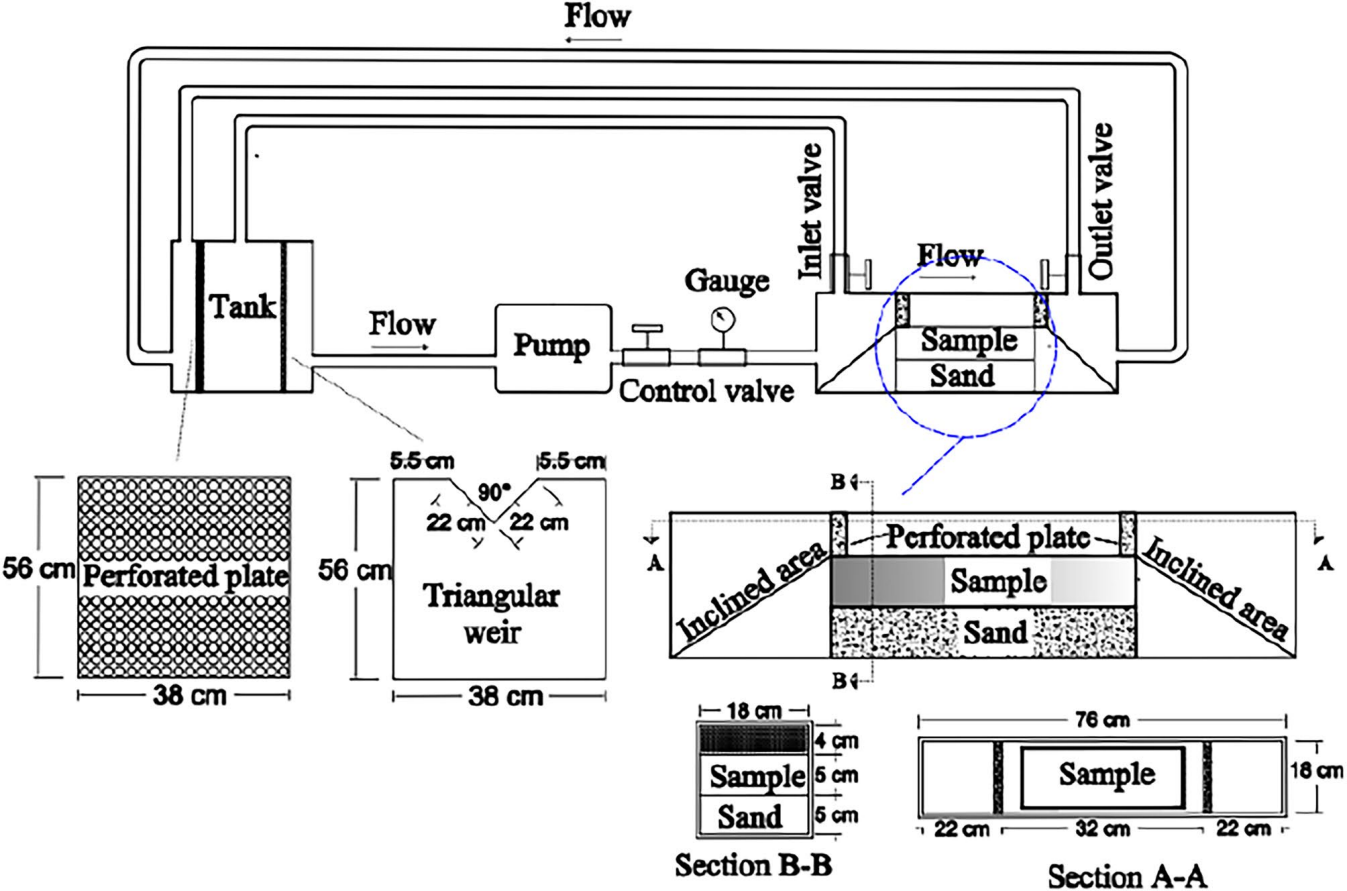
Schematic illustration of the velocity-base dissolution test apparatus.

This device was designed to simulate the conditions of the gypsum rock layers under water seepage with high velocity (discharge) in the dam abutments. Figure 6 represents the schematic of the velocity-base dissolution test apparatus. It comprises a Plexiglas container, a pump unit with associated parts, and a 120-L storage tank.

The Plexiglas container with a 76 cm length, 18 cm width, and 14 cm height is the testing vessel of the sample. The inlet and outlet of this container were inclined to minimize the turbulence flow of circulated water during the test. Additionally, a perforated plate was placed in both. The container has a Plexiglas cover with two inlet and outlet valves for air bubble removal, secured by a steel frame for integrity during the tests. To maintain stable temperatures throughout the test, the pump was separated from the electromotor. There is a perforated plate in the tank that prevents air bubbles during water circulation in the system. In this apparatus, the flow rate was measured using the V-notch method^50^.

Regarding the test preparation, firstly, a layer of pure sand was placed at the container base to allow the sample to reach the appropriate level at which the circulated water flows over its top surface. Next, the block of surface sample was placed on this sand layer. To ensure only the top surface of the sample was exposed to water flow, the sections touching the container inner wall were sealed with 1 cm thick silica glue. This prevented water leakage from coming into contact with the other surfaces beyond the top surface. Finally, the container was tightly covered, and 100 L of water were added to the tank. Now the sample was ready to be tested.

Using this apparatus, three untreated surface samples (S) were tested at different water flow rates of (0.07 m/s, 0.25 m/sec and 0.3 m/sec) and the treated surface samples (S-ACR and S-PU) were tested at 0.3 m/sec water flow rate (the highest flow velocity of the study), and at 25 ± 2 °C.

### Dissolution test using pressure-base apparatus

**Figure 7:**
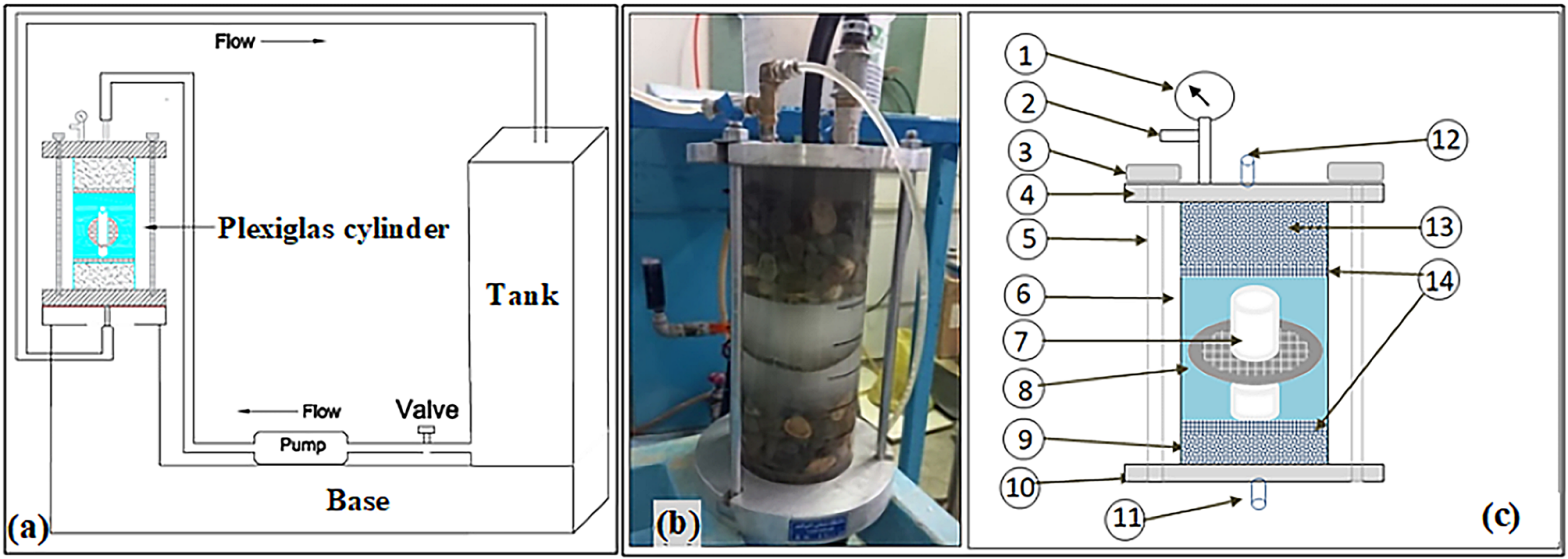
Pressure-base dissolution test apparatus and its main parts, (**a**) schematic illustration of the main parts, (**b**) image of equipped Plexiglas cylinder, and (**c**) schematic illustration of the equipped Plexiglas cylinder ; Note:(1) pressure gauge, (2) air vent, (3) screw, (4) top steel bench, (5) clamp, (6) Plexiglas cylinder, (7) rock sample, (8) grid , (9) lower gravel layer) (10) bottom steal bench, (11) outlet valve, (12) inlet pipe, (13) upper gravel layer, (14) perforated plate.

To simulate the conditions of gypsum rock layers under water pressure at the dam foundation, this apparatus was designed. The main units of this apparatus are: (a) a Plexiglas cylindrical vessel with a height of 30 cm and a constant internal diameter of 11 cm (b) a pump unit (c) a tank with a storage capacity of 250 L. Figure 7 illustrates the pressure-base dissolution test apparatus. As can be seen from the figure, at either end of the Plexiglas cylinder, there is a steel bench joined by three steel clamps, and screwed to prevent water leakage during testing. Each bench has its valve (inlet and outlet), which allow water circulation in the system, and an air vent is connected to the inlet valve to eliminate air bubbles. The configuration also incorporates a pressure gauge to monitor the testing pressure. The pump circulates the water between the tank and the Plexiglas cylinder vessel during the tests.

Regarding the test procedure, initially, all valves were closed. Subsequently, a 5 cm thick layer of gravel was placed at the bottom of the cylindrical container. A steel mesh was then placed over the gravel layer. After that, the core sample was positioned on this steel mesh. To remain the sample stable during the test, a grid was installed in the center of the sample. Next, another steel mesh plate was placed above the core sample, and a final layer of gravel 12 cm thick was added over the last steel mesh. Following this, the Plexiglas cylindrical vessel was secured using clamps and screws. The test preparation was finalized by adding 100 L of water to the tank.

The experimental steps started by opening the valves, followed by the incremental introduction of water at a subdued pressure from the tank into the container via the inlet valve until the container was entirely filled with water. Following this step, and verifying the lack of trapped air, the pump circulation rate was gradually raised through an inverter until the pressure of 400 kPa was gained. This setup enabled the pump to convey water from the tank into the container and subsequently circulate it back to the tank.

In this experiment, the untreated core samples labeled as G1, G2, G3, and G4 were tested for 48 h at a temperature of 25 ± 2 °C. Then, the sample with the highest dissolution rate (G2) was chosen for coating with grouts and further tests.

It is worth mentioning that, during the experiments, the water samples were collected at regular time interval to measure the concentration of dissolved gypsum in circulated water. The titration approach and inductively coupled plasma (ICP) were applied in the cases of untreated and treated samples, respectively.

### Other tests

Electrical conductivity (EC) of the water samples was measured using ATC (Automatic Temperature Compensation) equipped device.

In the dissolution tests on untreated rock samples, the EDTA titration method proposed by Horvai et al.^51^ was used to measure the dissolved gypsum content in the water samples. In this procedure, 50 ml of the water sample was measured with a pipette and poured into a clean conical flask. Then, 10 ml of a buffer (1 M NH4OH) solution was added to the flask to reach a pH of 10–11, followed by the addition of a Eriochrome Black T indicator.

The titration began with the gradual addition of the standardized EDTA (0.01 M Ethylenediaminetetraacetic acid) solution from a burette to the sample solution in the conical flask. The titration reached its endpoint as the solution changed color from red to blue, indicating the reaction between EDTA and calcium ions. The recorded EDTA volume was utilized to calculate the quantity of dissolved calcium using Eq. (4). To improve result accuracy, the test was done three times, and the mean of the results was taken.

4 $${Ca}^{+2} (mg/lit)=\frac{\text{E}\times \text{M}\times 40000}{\text{ water sampel }(\text{ml})}$$

where, E is the volume of EDTA and M is the molarity of EDTA (0.01).

In the dissolution tests on treated rock samples, Inductively Coupled Plasma (ICP) Spectroscopy was utilized to measure the calcium concentration in the water samples. This analytical test is utilized to measure and identify elements within a sample by assessing the ionization of elements present in the sample.

## Results and discussion

### Dissolution tests of the untreated surface samples (S) using velocity-base apparatus

**Figure 8:**
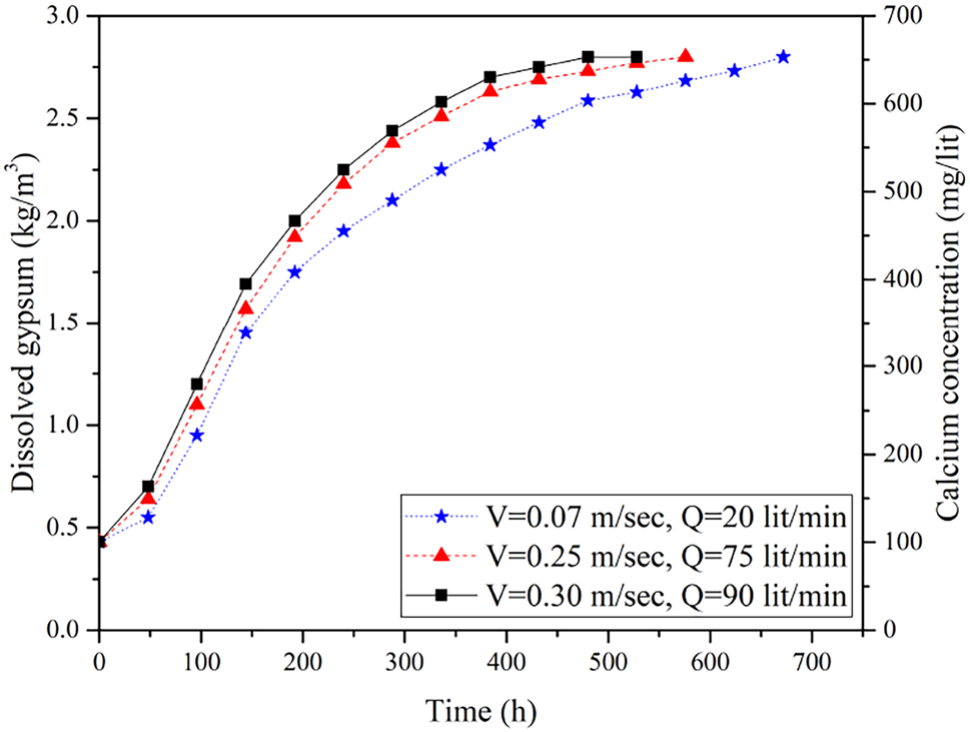
Variations of gypsum concentration over time in dissolution test of the untreated surface samples at different flow velocities.

Three untreated surface samples with identical chemical composition at different water velocities of (0.07 m/sec, 0.25 m/sec and 0.30 m/sec) were tested until reaching the saturation point of the solvent (2.8 kg/m^3^). Figure 8 shows the relationship between the quantities of dissolved gypsum versus time for the different water velocities.

It can be seen that all samples showed a similar increase in dissolved gypsum quantities over time, however, there was a noticeable difference in the saturation duration, reflecting the influence of different water velocities. Specifically, at a water velocity of 0.07 m/sec, the saturation point was achieved at 672 h; however, when the water velocities were 0.25 m/sec and 0.30 m/sec, the saturation duration reached 576 h and 528 h, respectively. This finding aligns with the results of Imam et al.^52^.

**Figure 9:**
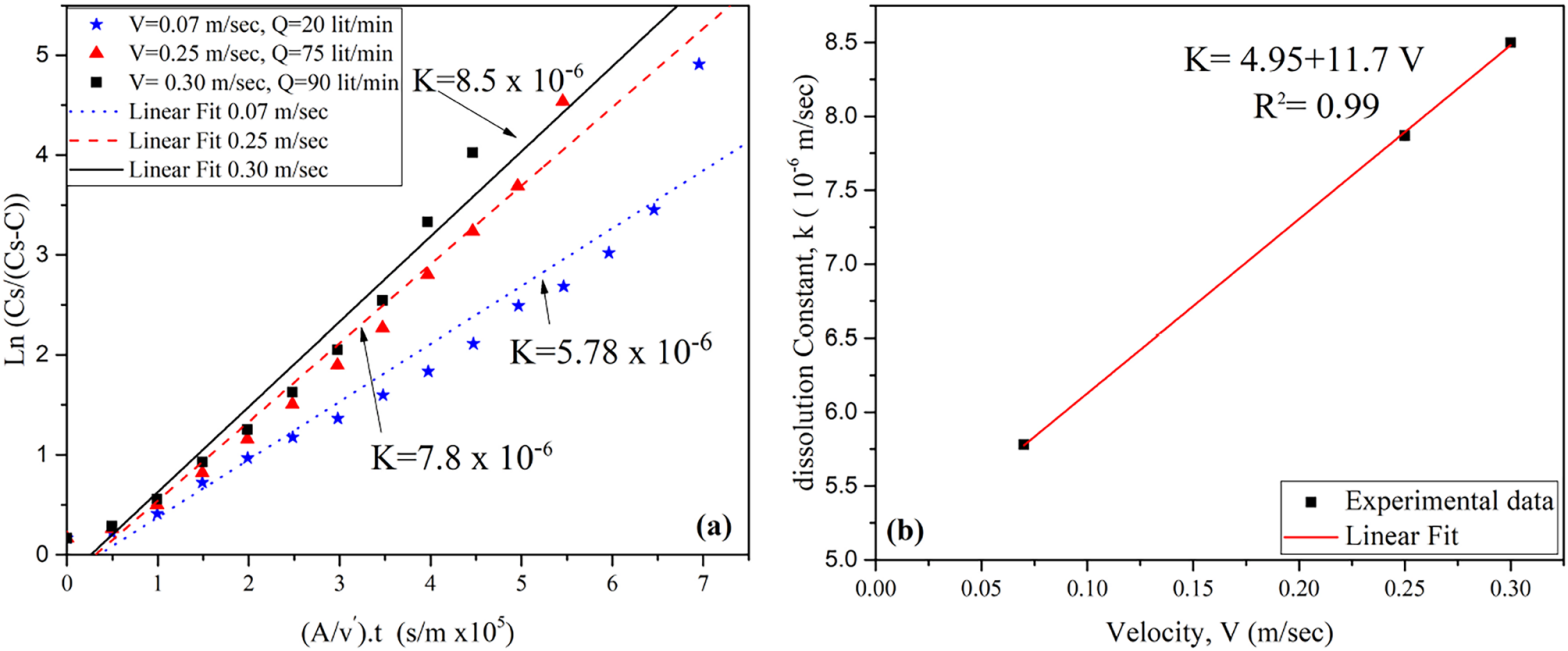
(**a**) Determination of the constant dissolution rate (k) of surface gypsum rock samples at different water flow velocities (V), (**b**) Relationship between k and V.

By utilizing Eq. (3), drawing a graphical relationship between $\left(\frac{{\text{C}}{\text{s}}}{{\text{C}}{\text{s}}\text{-C}}\right)$ and $\frac{\text{A}}{\text{v`}}{\text{t}}$, and determining the dissolution rate constant for each test, the correlation between dissolution rate and water flow velocity was further clarified. Figure 9 shows the effect of water flow velocity on dissolution rate constant.

As can be seen from Fig. 9a, when the velocity of circulated water was 0.07 m/sec, the value of K was 5.78 × 10^–6^, while the value of K reached 7.8 × 10^–6^ and 8.5 × 10^–6^ at the velocities of 0.25 m/sec and 0.30 m/sec, respectively. The variation of K was plotted as a function of water velocity in Fig. 9b. It can be noted that there is a linear relationship between K and V. Similar results were reported by James and Lupton ^15^.

It is noteworthy that the velocity with the highest dissolution rate (0.3 m/sec) was used in testing the treated samples with ARC and PU.

**Figure 10:**
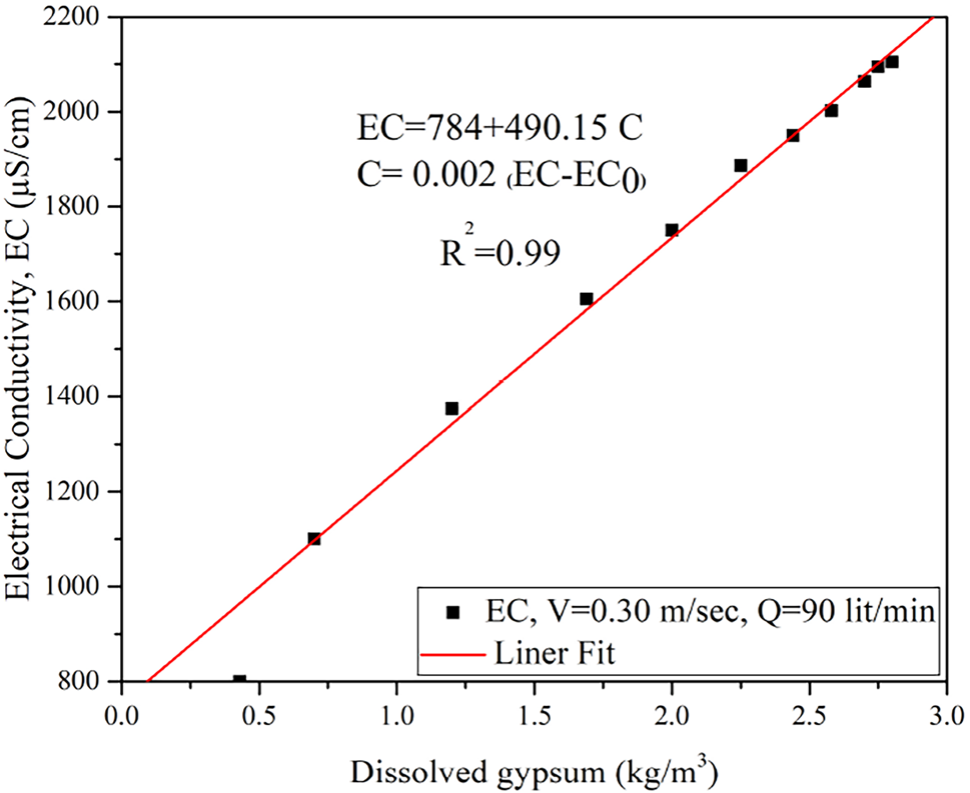
Variations of EC with gypsum concentration for flow velocity of 0.3 m/sec.

The electrical conductivity (EC) of tap water and saturated water samples were 800 μS/cm to 2150 μS/cm, respectively. This technique was used as an index test before titration and ICP tests of the water samples. Minor variations in the EC results were due to the presence of other ions in the water samples. Figure 10 shows the calcium (gypsum) concentration and EC relationship for the untreated sample tested under 0.3 m/s water flow.

### Dissolution tests of the core samples (G) using pressure-base apparatus

**Figure 11:**
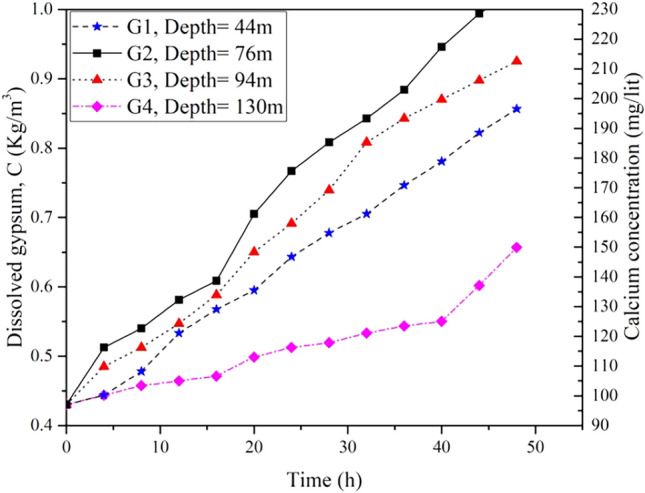
Variation of quantities of dissolved gypsum over 48 h for untreated borehole core samples under constant pressure of 400 kPa.

Four untreated core samples (G1 to G4) with different chemical compositions (gypsum and anhydrite) were tested under a constant pressure of 400 kPa for 48 h. Figure 11 demonstrates the graphical relationship between the quantities of dissolved gypsum over time.

It can be observed that G2 has displayed the most rapid dissolution rate, whereas G4 showed the minimum dissolution rate throughout the testing period. This distinction can be attributed to variations in the chemical composition of the respective samples. Based on the chemical analysis finds, G2 is predominantly composed of gypsum, while G4 is primarily constituted of anhydrite. This finding agrees with the result of Zanbak and Arthur^53^, they concluded that under water pressure conditions, anhydrite exhibits lower solubility compared to gypsum.

Notably, the sudden changes in the slopes of the curves for sample G4 at t = 40h, and G2 at t = 17h may be attributed to the weakened effect of the water on the structural integrity of the samples over time, consequently, the samples have experienced surface or internal fractures or structural weaknesses that accelerated dissolution rate.

It is noteworthy that among the tested core samples, the sample with the highest dissolution rate (G2) was selected for additional study after being treated with ARC and PU.

### Dissolution test of the ARC treated gypsum rock samples

**Figure 12:**
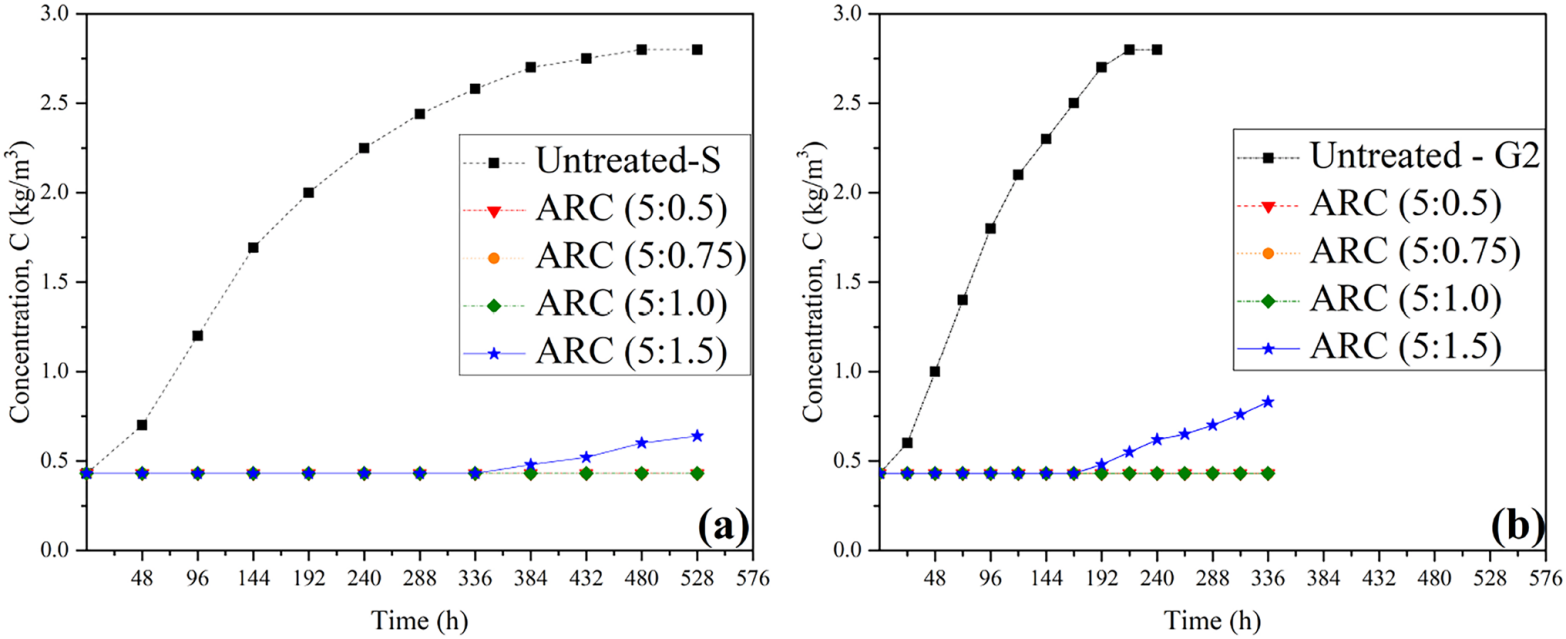
Effect of ARC grouts on the solubility of gypsum rock samples with respect to different cement contents, (**a**) surface samples under 0.3 m/sec flow water rate, (**b**) core samples under 400 kPa pressure.

Fig. 12 illustrates the effect of ARC on the gypsum rock solubility at the highest flow velocity of the study (i.e., V = 0.3 m/s) (Fig. 12a), and under 400 kPa water pressure (Fig. 12b).

The observed trends for all ARC coated samples except (1.5 percent of cement content) indicated a cessation in gypsum dissolution at the test duration. Without ARC grout, the concentration of dissolved gypsum under high velocity and 400 kPa pressure reached the saturation state of 2.8 g/L over 528 h and 240 h, respectively. While during these periods for testing the treated samples with different ratios of (acrylic: cement 5:0.5, 5:0.75, and 5:1), the gypsum concentration of the circulated water remained at the original value of 0.43 g/L (Ca^+2^ concentration of the used water). This halting in dissolution can be attributed to the presence of ARC, which acted as a robust film, coated the surface of the gypsum rock and isolated the rock from the influence of water. According to the study conducted by Priyadharshini and Ramakrishna^54^, when the water evaporates during the setting time of the acrylic grout, the resin particles are attracted together and then agglomerate to form a film. The forces responsible for the merging of the polymer particles stem from both surface tension and capillary forces. Acrylic aided a dual purpose: it enhanced adhesion between Portland cement and the gypsum rock, and reduced the likelihood of shrinkage and grout cracking by creating a more flexible structure with lower stiffness, this result confirmed by Mullenax^55^ and Akinyemi and Omoniyi^56^. Additionally, as reported by Jiang et al.^57^, acrylic polymer not only impart flexibility to polymer cement mixture but also function as cementitious phases.

However, beyond the certain dosage of acrylic- cement of (5:1) ratio, specifically in the (5:1.5) ratio, the coated samples after 336 h under water velocity and after 168 h under pressure of 400 kPa began dissolution. These consequences showed the optimum dosage of ARC to improve the gypsum rocks. The resurgence of dissolution in the treated gypsum can be primarily attributed to the increase in Portland cement content with the presence of aluminum sulfate accelerator in the grout which reduced the setting time and enhanced drying shrinkage of the grout. This result was also confirmed by Kan et al.^58^, who investigated the impact of aluminum sulfate on Portland cement. Also, the cracking phenomenon in grouts due to the content of more than the optimal ratio of cement has been confirmed by Akinyemi and Omoniyi^56^. On the other hand, the starting of the dissolution process in the (5:1.5 ratio) ARC treated sample showed the resistance of the ARC grout to hydrolysis, in other words, it revealed the influence of both factors of water velocity and pressure on the grout. This result aligns with the outcome obtained by Momber and Kovacevic^59^; They concluded that high-velocity water flow leads to erosion of concrete, which is primarily driven by the formation and propagation of microcracks.

ARC with different cement contents from 0.5 to 1.0 percent has effectively hindered the solubility of gypsum over a given duration. The variation in these ratios impacts the setting time of the grouts. The selection of the most suitable percentage of acrylic to cement is contingent upon the target of the grouting.

### Dissolution test of the PU treated gypsum rock samples

**Figure 13:**
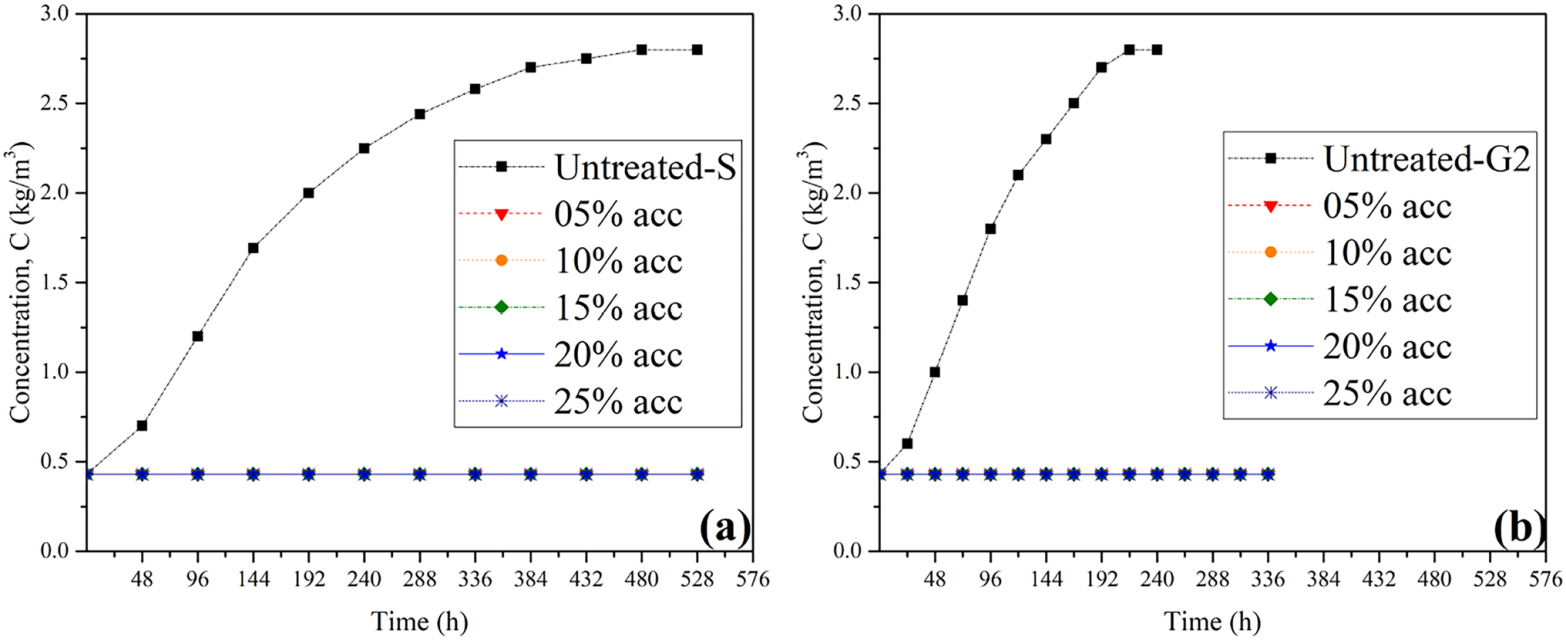
Dissolution test for the untreated and the polyurethane (PU) coated gypsum samples with respect to various accelerator additions (different density foams), (**a**) Surface samples under 0.3 m/sec flow water rate, (**b**) core samples under 400 kPa pressure.

Fig. 13 compares the results of a series of dissolution tests conducted on both untreated and PU treated samples at the highest flow velocity of the study (i.e., V = 0.3 m/s) (Fig. 13a), and under 400 kPa water pressure (Fig. 13b). As evident from the results, PU demonstrates a substantial positive effect on halting the gypsum sample solubility.

As the untreated gypsum curve exhibited a consistent upward trend in dissolution, the curve representing the PU coated samples remained stable at the original concentration (tap water salinity) for all various PU/acc ratios (Due to the similarity of the treated samples results, the curves of the different tests are shown as a single curve in the Fig. 13a,b. Furthermore, although the density of the grout decreases with increasing accelerator content, and consequently the expansibility of the gelled PU increases, interestingly, test results for all accelerator contents (from 5 to 25% accelerator) showed similar outcomes, which was the prevention of the gypsum dissolution during the testing period. This can be attributed to the strong resistance of the PU to hydrolysis and erosion. Similar outcome was obtained by Jacob^37^, who concluded that the durability of polymer adhesives in the presence of water is attributed to its high resistance to hydrolysis. Additionally, the used PU in this research represents the hydrophobic type of PU, according to the studies by Vipulanandan et al.^31^ and Buzzi et al.^60^, hydrophobic materials on the foam surface create a water-repellent barrier, and significantly prevent water absorption due to the combination of surface repellency and the closed-cell structure (the cells are not interconnected). Therefore, water is unable to permeate the foam, and the closed-cell design minimizes the total moisture content.

Furthermore, the high resistance of PU against both water pressure and high velocity water flow, simultaneously the lack of gap between PU and the rock sample during the whole time of the test further indicates the strong adhesion between PU (with different density foams) and the gypsum samples. This result is consistent with the result of Saunders and Frisch^61^, who concluded that PU is a coating material with excellent adhesion to various substances.

When their waterproof capacity of the materials is compared, as seen fromFig. 14, the PU is more impactful grout compared to the ARC. PU foam with different densities (different accelerator dosage) completely stopped the dissolution process under both factors. In contrast, ARC was able to achieve the similar result, but only when optimized at specific ratios of (5: 1.0).

**Figure 14:**
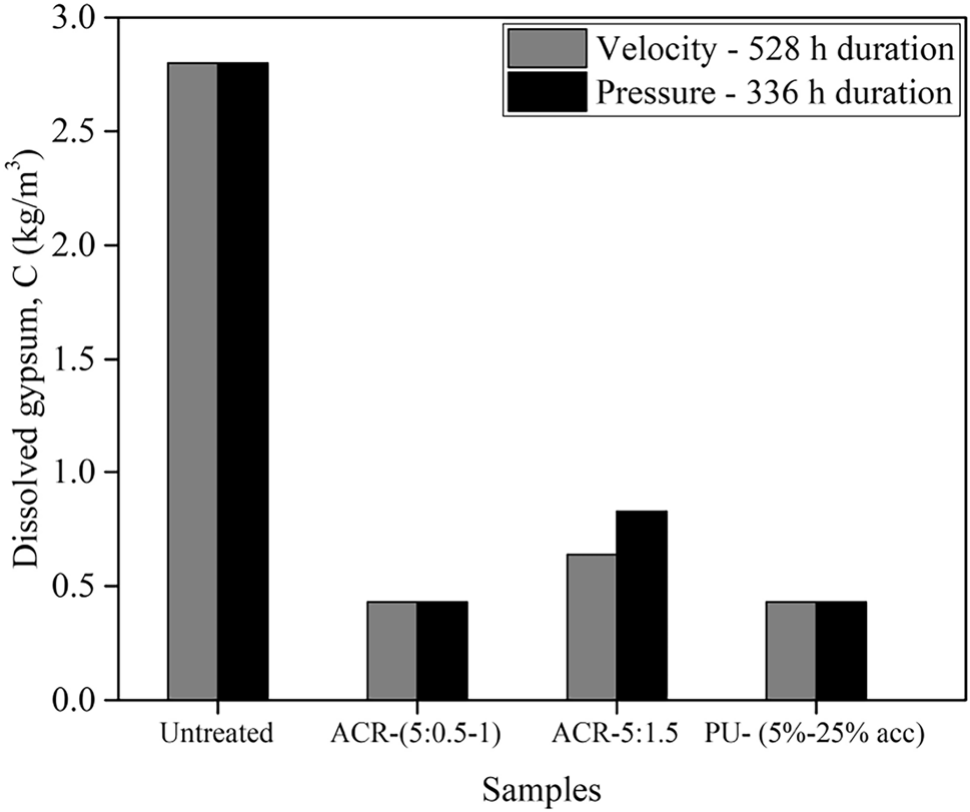
Effect both ARC and PU on the gypsum solubility at velocity 0.3 m/sec and under pressure 400 kPa.

Nevertheless, there exist essential distinctions between the two types of grouts. ARC lacks a quick setting, whereas the gelling time of PU is quicker and to some extent controllable. additionally, compared to ARC, PU is more penetrable and can fill much smaller cracks^25,26^, however it is more difficult to use underground because its behavior (expansibility and quick gelling time) cannot be controlled underground^60^. While ARC is more stable in different environments, nevertheless, there are defects with ARC utilization, particularly in its capacity to penetrate very fine soils or reach the narrow cracks and fissures within rocks^62^. On the other hand, in terms of cost, ARC is more economical compared to PU.

## Conclusions

The purpose of the current study was to overcome the solubility of gypsum rocks using chemical grouts. The used rock samples were taken from the surface gypsum rocks and problematic layers of brecciated gypsum situated at varying depths of the Mosul Dam foundation. In summary, the main results derived from this simulated experimental investigation can be summarized as follows:

•Under both factors of water velocity and pressure, Except for the case involving 1.5 percent of cement, the (ARC) grout has a significant effect in controlling the dissolution of soluble rocks, and plays a vital role in attaining full hydrolysis resistance under the influence of both water velocity and water pressure factors. Beyond the optimal ratio of 5:1, with increasing cement content the sealing strength of the ARC grout decreases, indicating that using cementitious grout alone cannot yield satisfactory results in treating gypsum rocks. This reveals the defect in the protective measurement of the Mosul Dam foundation, due to the use of cement and certain additives during the grouting. It also validates the source of persistent seepage at the Mosul Dam.
•The water pressure resistance of ARC is lower in comparison to its resistance against water flow velocity. The sample coated with ARC at a ratio of 5:1.5 experienced dissolution after 336 h under high-velocity water flow and after 168 h under pressure of 400 kPa.
•All PU coated gypsum samples with different accelerator quantity (different density) resulted in halting of dissolution during the test under both factors of water velocity and pressure. The lack of gap between PU and the rock sample during the whole time of the test further indicates the strong adhesion between PU and the gypsum samples.
•From a perspective of sealing strength, PU proves to be a more effective choice than ARC. This is evident in the PU treated gypsum samples; they did not experience any dissolution during the given time, whereas the sample coated with acrylic/cement ratio of 5:1.5 exhibited dissolution during the experiments.

## Data Availability

The data generated and used during the current study are available from the corresponding authors by request.

## Abbreviations

*A*: Surface area, m^2^
*C*: Concentration, kg/m^3^
*C*_*s*_: Saturated concentration (solubility), Kg/m^3^
EC: Electrical conductivity, μS/cm
*K*: Dissolutions rate constant, m/s
*m*: Dissolved mass, kg
t: Time, hour
v`: Water volume, L
*V*: Water velocity, m/s

## References

[1] Wakeley, L.D., Kelley, J.R. & Pearson, M.L. Geologic conceptual model of Mosul Dam. (2007).

[2] Adamo N, Al-Ansari N, Laue J, Knutsson S, Sissakian V (2017). Risk management concepts in dam safety evaluation: Mosul Dam as a case study. J. of Civil Eng. Arch..

[3] Al-Ansari N, Adamo N, Knutsson S, Laue J, Sissakian V (2020). Mosul dam: Is it the most dangerous dam in the world?. Geotech. Geol. Eng..

[4] Lykoshin, A., Molokov, L. & Parabutchev, I. Karst and dam engineering. *Russian.“Gidroproekt”, Moskva (in Russian)* (1992).

[5] Johnson, K. Problems of dam construction in areas of gypsum karst. *Кapcтoвeдeниe XXI вeк: тeopeтичecкoe и пpaктичecкoe знaчeниe* 236–240 (2004).

[6] Gutiérrez F, Mozafari M, Carbonel D, Gómez R, Raeisi E (2015). Leakage problems in dams built on evaporates. The case of La Loteta Dam (NE Spain), a reservoir in a large karstic depression generated by interstratal salt dissolution. Eng. Geol..

[7] Hu, W. Study on the Formation of Triassic' Gypsum-Dissolved-Strata' in Guizhou Province and the Seepage Prevention for Reservoirs. In *Karst Hydrogeology and Karst Environment Protection. Proceedings 21st IAH Conference.* (ed. Yuan, D.) Vol. 2 (IAHS-AISH Publication, Guilin, China, 1988).

[8] Giambastiani, M. Geomechanical characterization of evaporitic rocks. *Soft R. Mech. Eng.* 129–161. 10.1007/978-3-030-29477-9_6 (2020).

[9] Nernst W (1904). Theorie der Reaktionsgeschwindigkeit in heterogenen Systemen. Z. Phys. Chem..

[10] Maslov, N. & Naumenko, V. Stability conditions of hydro-structures foundation at rocks containing gypsum. *Dissolution and Disintegration of Rocks*, 71–81 (1957).

[11] Fabuss, M., Korozi, A., Middleton, T. & DeMonico, J. Office of saline water rep. *Contract*, 14-01-0001–1269 (1969).

[12] Wigley T (1971). Ion pairing and water quality measurements. Can. J. Earth Sci..

[13] Milanović P, Maksimovich N, Meshcheriakova O (2019). Dams and reservoirs in evaporites.

[14] Shearman, D. & Mossop, G. Origins of secondary gypsum rocks. *Bullet. Instit. Mining Metal.* **82**, B147–B154 (1973).

[15] James A, Lupton A (1978). Gypsum and anhydrite in foundations of hydraulic structures. Geotechnique.

[16] James, A.N. *Soluble materials in civil engineering.* (Ellis HOIwood, Chichester, 1992).

[17] Nikolaev, A. & Foregina, E. *Protective effect of films on gypsum.* (Protective Films on Salts (in Russian). Izd. Akad. Nauk SSSR, Moscow, Leningrad, 1944).

[18] Fattah MY, Al-Ani MM, Al-Lamy MT (2014). Studying collapse potential of gypseous soil treated by grouting. Soils Found..

[19] Faris, M.K. Evaluating the Stabilization of Gypseous and Gypsiferous Sands Using Different Chemical Additives to Mitigate Gypsum Dissolution. Doctoral dissertation University of South Carolina (2020).

[20] Buggakupta W, Tounchuen K, Panpa W, Jinawath S (2020). Early production of high strength and improved water resistance gypsum mortars from used plaster mould and cullet waste. J. Mater. Civ. Eng..

[21] Adamo N, Al-Ansari N (2016). Mosul dam the full story: Engineering problems. J. Earth Sci. Geotech. Eng..

[22] Karol RH (2003). Chemical grouting and soil stabilization, revised and expanded.

[23] Allen G, Bevington JC, Eastmond GC (1989). Comprehensive Polymer Science Chain Polymerization I.

[24] Weaver KD (2007). & Bruce.

[25] Li L (2010). Experimental study of a new polymer grouting material. Chin. J. R. Mech. Eng..

[26] Kolay PK, Dhakal B, Kumar S, Puri VK (2016). Effect of liquid acrylic polymer on geotechnical properties of fine-grained soils. Int. J. Geosynth. Ground Eng..

[27] Cinar A, Maerz N, Cinar A (2020). An experimental study of chemical grouting materials for optimum mechanical performance. Geo-Congress 2020.

[28] Golhashem MR, Uygar E (2020). Volume change and compressive strength of an alluvial soil stabilized with butyl acrylate and styrene. Constr. Build. Mater..

[29] Pakir F, Marto A, Yunus NM, Tajudin SAA, Tan CS (2015). Effect of sodium silicate as liquid based stabilizer on shear strength of marine clay. J. Teknol..

[30] Bhardwaj AK, McLaughlin RA, Levy GJ (2010). Depositional seals in polyacrylamide-amended soils of varying clay mineralogy and texture. J. Soil. Sed..

[31] Vipulanandan C, Kazez MB, Henning S (2012). Pressure-temperature-volume change relationship for a hydrophilic polyurethane grout. Grout. Deep Mix..

[32] Wang C (2020). An experimental study on the reinforcement of silt with permeable polyurethane by penetration grouting. Adv. Civil Eng..

[33] Saleh S, Ahmad K, Mohd Yunus NZ, Hezmi MA (2020). Evaluating the toxicity of polyurethane during marine clay stabilisation. Environ. Sci. Pollut. Res..

[34] Sabri M, Bugrov A, Panov S, Davidenko V (2018). Ground improvement using an expandable polyurethane resin. MATEC Web of Conf..

[35] Davies P, Evrard G (2007). Accelerated ageing of polyurethanes for marine applications. Polym. Degr. Stabil..

[36] Bayer, O. The Diisocyanate Polyaddition Process. *Angew. Chem*. A, **59**, 275 (1947).

[37] Jacob A (2006). The popularity of carbon fibre. Reinf. Plastics.

[38] León, L.R. & Matthews, C. Storm water quality handbooks: construction site best management practices (BMPs) manual (2003).

[39] Khelifa F, Habibi Y, Dubois P, Khelifa F (2016). Nanocellulose-based polymeric blends for coating applications. Multifunctional polymeric nanocomposites based on cellulosic reinforcements.

[40] Chhun K-T, Lee S-H, Keo S-A, Yune C-Y (2019). Effect of acrylate-cement grout on the unconfined compressive strength of silty sand. KSCE J. Civil Eng..

[41] Adamo N, Al-Ansari N, Sissakian V, Laue J, Knutsson S (2019). Mosul Dam: Geology and safety concerns. J. Civil Eng. Arch..

[42] Sissakian VK, Adamo N, Al-Ansari N (2020). The role of geological investigations for dam siting: Mosul Dam a case study. Geotech. Geol. Eng..

[43] Wheeler, A. Mosul Dam assessment-Report on Site Visit. *Baghdad. Iraq* (2004).

[44] Al-Ansari, N., Barazanji, A., Al-Jabari, M. & Gayara, A. Geological Investigation of Mosul Dam Site. *Cofidential Report* (1984).

[45] Wheeler, M. et al. Mosul Dam assessment, review of 1984 dam break and flood wave study for Mosul Dam, Iraq. *Black and Veatch, UK* (2004).

[46] Sissakian VK, Al-Mousawi HA (2007). Karstification and related problems, examples from Iraq. Iraqi Bull. Geol. Min..

[47] Koukouzas N, Vasilatos C (2008). Mineralogical and chemical properties of FGD gypsum from Florina, Greece. J. Chem. Technol. Biotechnol..

[48] Al-Jaroudi SS, Ul-Hamid A, Mohammed A-RI, Saner S (2007). Use of X-ray powder diffraction for quantitative analysis of carbonate rock reservoir samples. Powder Technol..

[49] Saha M (2005). Effect of density, microstructure, and strain rate on compression behavior of polymeric foams. Mater. Sci. Eng. A.

[50] Bos, M.G. Discharge measurement structures. Ilri (1976).

[51] Horvai GG, Christian D (2014). Purnendu (Sandy) Dasgupta and Kevin Schug: Analytical chemistry.

[52] Imam, R., Mahmoudi, A. & Farsghoshooni, A. Experimental study of gypsum and anhydrite dissolution due to surface flow. *Proceedings of the GeoEdmonton* (2018).

[53] Zanbak C, Arthur RC (1986). Geochemical and engineering aspects of anhydrite/gypsum phase transitions. Bull. Assoc. Eng. Geol..

[54] Priyadharshini, S. & Ramakrishna, G. Strength and durability evaluation of latex modified sisal fibre reinforced concrete. *Int. J. Res. Sci.* **8**, 9–16 (2014).

[55] Mullenax PD (1984). Acrylic polymer Portland cement coating composition.

[56] Akinyemi BA, Omoniyi TE (2017). Engineering properties of acrylic emulsion polymer modified bamboo reinforced cement bonded composites. Eng. Struct. Technol..

[57] Jiang YJ, Li L, Wang HS, Wang R, Tian Q (2015). Influence of acrylic emulsion on polymer-cement waterproof coating. Adv. Mater. Res..

[58] Kan CY, Lan MZ, Kong LM, Yang JB (2013). Effect of aluminium sulfate on cement properties. Mater. Sci. Forum..

[59] Momber A, Kovacevic R (1994). Fundamental investigations on concrete wear by high velocity water flow. Wear.

[60] Buzzi O, Fityus S, Sasaki Y, Sloan S (2008). Structure and properties of expanding polyurethane foam in the context of foundation remediation in expansive soil. Mech. Mater..

[61] Saunders H, Frisch K (1964). Chemistry and technology, part II: technology.

[62] Sögaard C, Funehag J, Abbas Z (2018). Silica sol as grouting material: a physio-chemical analysis. Nano Conver..

